# What is the psychosocial burden of COVID‐19 on people with pulmonary hypertension?

**DOI:** 10.1111/crj.13570

**Published:** 2022-12-20

**Authors:** Gregg H. Rawlings, Andrew R. Thompson, Iain Armstrong, Nigel Beail

**Affiliations:** ^1^ School of Social Sciences Nottingham Trent University Nottingham UK; ^2^ South Wales Clinical Psychology Training Programme Cardiff University Cardiff UK; ^3^ Sheffield Pulmonary Vascular Disease Unit, Royal Hallamshire Hospital Sheffield Teaching Hospitals NHS Foundation Trust Sheffield UK; ^4^ Clinical and Applied Psychology Unit University of Sheffield Sheffield UK

To the Editor,

People with pulmonary hypertension (PH) are more vulnerable to the SARS‐CoV‐2 virus; for example, greater severity of COVID‐19 symptoms and higher rates of mortality having been observed in this clinical group.^1^, ^2^ To better understand the psychosocial impact of the pandemic on individuals with PH and their approach to coping,^3^, ^4^, ^5^ we interviewed individuals in July 2020 who were recruited from Pulmonary Hypertension Association UK. Data were analysed using directed content analysis.^6^ Ethical consent was obtained from the Department of Psychology at the University of Sheffield (035318).

Overall, 121 participants took part; mean age 58.3 years, 74% were female and 99% self‐reported as White. Participants were diagnosed with PH for an average of 8.5 years. Nearly two thirds had either idiopathic PH or chronic thromboembolic PH, with almost half self‐reporting their WHO functional class as II or III.^7^

Table 1 shows examples of key themes that emerged from participants' responses. Many losses were described; most common, and for some the hardest part, seems to have been lost connections with friends and families and being unable to leave their homes to socialise, for pleasure or daily chores and work: ‘Essentially, my life as I know and love it no longer exists’. Individuals described a reduction in their independence, having to ask other people to ‘do certain things’, which made them feel ‘weak’. All of this was linked to a deterioration in health, as well as an increase in PH symptoms. Low mood, anxiety, loneliness, reduced motivation and self‐confidence were all experienced.

**TABLE 1:** Example of key themes

	Theme	*n*
Most difficult part of the COVID‐19 pandemic	Not being able to see loved ones and others, loneliness and feeling isolated	60
	Being able to socialise, missing out on normal life, feeling ‘forgotten’ about, getting on with life and loss of freedom	46
	Catching the virus and dying or their respiratory difficulties becoming worse as a result	19
	Declining emotional and physical well‐being, such as change in mood, low motivation and losing confidence	13
	Uncertainty and lack of information about the virus	10
	Other people being inconsiderate or not following COVID‐19 guidance	10
	Potential risk or actual change to health or social care resources	8
	Increased dependency (sense of loss of independence)	8
	Seeing how others have been impacted by the pandemic	6
	Own sense of safety	2
	Working from home	1
	‘Nothing’	1
	Reduction of positive ways of coping with PH such as distraction	1
	Increase costs associated with shopping online	1
	Needing to tell other people they have PH	1
	Relaxing of the infection control rules too early	1
Most helpful ways of coping	Keeping busy and in a routine, going into nature and distraction such as the internet or learning a new skill	57
	Contact with others and spending time with loved ones	42
	Practical support, that is, help with shopping, and information on the virus	15
	Avoiding other people (due to infection control) and stresses such as ‘switching the news off’	11
	Support from healthcare services, services for mental health and PH associations	11
	Engaging in physical activity	10
	Following infection control rules	6
	‘Nothing’	8
	Sleeping	1
	Faith	1

There was a general sense that participants felt they have lost part of their life having to shield for so long and somewhat ‘cut off’ or left behind by society. Participants expressed anxiety over integrating ‘back into a, frankly, frightening community’. This fear also stemmed from other people failing to take infection control guidelines ‘seriously enough’. Individuals were concerned over social rejection due to their own views toward infection control. There was an impression that some felt guilty as their health risks imposed upon others. Loss of perceived safety was reported with participants uncertain of knowing when or how this would be returned. This resulted in feelings of ‘helpless’ and for the first time, ‘a person with an actual illness’. Uncertainty seemed to be, in part, perpetuated by inconsistent or lack of information about the virus and PH and the government's response. Overall, the pandemic has added to the burden of living with PH. Unlike most who find some comfort in knowing they may only experience mild symptoms, if any, those with PH expressed ‘worrying excessively that I will die’.

Friends, family, employers, volunteers and key workers were important sources of support: ‘They [family and friends] were what got me through my diagnosis [of PH] last year’. Contact with healthcare services and organisations, or just knowing they are there, was valuable. Participants described using technology to help manage feelings of isolation and maintain relationships. Website and apps provided participants with additional strategies to cope with difficult emotions and prevent rumination or ‘overthinking it all’. Technology also alleviated boredom and facilitated accessing advice on mental health and achieving daily chores. Distractions and developing new hobbies were common ways of coping; however, this often involved sedentary‐based activities. Denial, keeping busy, alcohol and avoidance were also described. Potentially more adaptive approaches were discussed such as going outdoors, acceptance, staying positive and volunteering. There was a general sense that following guidelines provided participants with a sense of control and safety, although could also fuel anxiety.

It was not our aim to provide an exhaustive overview of the difficulties this group have experienced. For starters, based solely on demographics, the voices of many are underrepresented. Our findings are consistent with the wider literature demonstrating the effects of the initial stages of the pandemic, including lower physical activity, sleep quality^8^ and mental health difficulties including anxiety and depression.^9^ Continued efforts are needed to investigate the ongoing burden on this group^10^, ^11^—and their carers for whom there is very little research^12^—in addition to their already heightened risk of experiencing difficulties associated with life with PH. This should include developing a greater understanding of the impact of the condition and how patients are coping in light of COVID‐19,^13^ routine screening for psychosocial difficulties and addressing these in care plans and creating accessible and PH‐specific interventions targeting common mental health and social problems in this clinical group.^14^

## CONFLICT OF INTEREST

The authors have no conflict of interests to declare.

## ETHICS STATEMENT

Ethical consent was obtained from the Department of Psychology at the University of Sheffield (035318).

## AUTHOR CONTRIBUTIONS

Gregg H. Rawlings was responsible for the conception of this article, data collection and analysis and writing the report for publication. He approved the final version for publication. Andrew R. Thompson made substantial contributions to research design and provided feedback on data analysis and final report. He approved the final version for publication. Iain Armstrong made contributions to data analysis and provided feedback on the final report. He approved the final version for publication. Nigel Beail made substantial contributions to research design and provided feedback on data analysis and final report. He approved the final version for publication.

## Data Availability

The data that support the findings of this study are available on request from the corresponding author. The data are not publicly available due to privacy or ethical restrictions.
